# TGFβ-Associated Signature Predicts Prognosis and Tumor Microenvironment Infiltration Characterization in Gastric Carcinoma

**DOI:** 10.3389/fgene.2022.818378

**Published:** 2022-05-18

**Authors:** Siyuan Liu, Zhenghao Li, Huihuang Li, Xueyi Wen, Yu Wang, Qilin Chen, Xundi Xu

**Affiliations:** ^1^ Hunan Provincial Key Laboratory of Hepatobiliary Disease Research and Division of Hepato-Biliary-Pancreatic Surgery, Department of General Surgery, The Second Xiangya Hospital, Central South University, Changsha, China; ^2^ Department of Urology Surgery, Xiangya Hospital, Central South University, Changsha, China; ^3^ Department of General Surgery, The South China Hospital, Shenzhen University, Shenzhen, China

**Keywords:** TGFβ, prognosis, tumor microenvironment, gastric carcinoma, immunotherapy

## Abstract

**Background:** Gastric carcinoma (GC) is a carcinoma with a high incidence rate, and it is a deadly carcinoma globally. An effective tool, that is, able to predict different survival outcomes for GC patients receiving individualized treatments is deeply needed.

**Methods:** In total, data from 975 GC patients were collected from TCGA-STAD, GSE15459, and GSE84437. Then, we performed a comprehensive unsupervised clustering analysis based on 54 TGFβ-pathway-related genes and correlated these patterns with tumor microenvironment (TME) cell-infiltrating characteristics. WGCNA was then applied to find the module that had the closest relation with these patterns. The least absolute shrinkage and selection operator (LASSO) algorithm was combined with cross validation to narrow down variables and random survival forest (RSF) was used to create a risk score.

**Results:** We identified two different TGFβ regulation patterns and named them as TGFβ Cluster 1 and Cluster 2. TGFβ Cluster 1 was linked to significantly poorer survival outcomes and represented an inflamed TME subtype of GC. Using WGCNA, a module (magenta) with the closest association with the TGFβ clusters was identified. After narrowing down the gene list by univariate Cox regression analysis, the LASSO algorithm and cross validation, four of the 243 genes in the magenta module were applied to build a risk score. The group with a higher risk score exhibited a considerably poorer survival outcome with high predictive accuracy. The risk score remained an independent risk factor in multivariate Cox analysis. Moreover, we validated this risk score using GSE15459 and GSE84437. Furthermore, we found that the group with a higher risk score represented an inflamed TME according to the evidence that the risk score was remarkably correlated with several steps of cancer immunity cycles and a majority of the infiltrating immune cells. Consistently, the risk score was significantly related to immune checkpoint genes and T cell–inflamed gene expression profiles (GEPs), indicating the value of predicting immunotherapy.

**Conclusions:** We have developed and validated a TGFβ-associated signature, that is, capable of predicting the survival outcome as well as depicting the TME immune characteristics of GC. In summary, this signature may contribute to precision medicine for GC.

## Introduction

Gastric carcinoma (GC) has the fifth highest incidence rate of cancer and the third highest fatality rate globally, causing approximately 723,000 deaths in 2012 (Smyth et al., 2020). Although classic therapies such as chemotherapy and even biological agents are utilized, patients with advanced gastric carcinoma still face an unsatisfactory prognosis. Immune checkpoint inhibitors (ICIs) especially programmed cell death-1 (PD-1) together with its ligand (PD-L1 or B7-H1), have been shown to be essential for the progression of several solid cancers and are likely to become a promising therapeutic target in gastric carcinoma treatment (Coutzac et al., 2019). Several clinical tests evaluating anti-PD-1/PD-L1-based curative methods as both first and later-line treatments have already produced inspiring results (Kang et al., 2017; Fuchs et al., 2018; Janjigian et al., 2018). However, not all patients in this cohort responded to ICI treatment. The primary predictors for immunotherapy effectiveness are likely to be PD-L1 expression together with defects in mismatch repair genes resulting in a microsatellite instability (MSI-H) phenotype (Vrána et al., 2018). All these biomarkers have failed for widespread clinical application because of unsatisfactory predictive accuracy or complex detection methods (Crispen and Kusmartsev, 2020; Hu et al., 2021). There is an urgent need to find robust biomarkers that could provide clues regarding the prognosis and immunotherapy response in GC.

The tumor microenvironment (TME) includes various cell types (fibroblasts, endothelial cells, immune cells, etc.) together with extracellular elements (growth factors, cytokines, extracellular matrix, hormones, etc.) that surround tumor cells and are nourished by a microvascular network (Wu and Dai, 2017). The TME can be generally divided into cold (non-T cell inflamed) and hot (T cell inflamed) conditions, which are mainly based on the production levels of proinflammatory cytokines and the infiltration degree of T cells (Gajewski, 2015). Hot tumors are characterized by T cell infiltration together with immune activated molecular signatures, whereas cold tumors are characterized by T cell exclusion or absence. Generally, more patients in the hot tumor subgroup will respond to immunotherapy, such as ICI therapy (Liu et al., 2020a). Transforming growth factor *β* (TGFβ) is a crucial enforcer of immune homeostasis and tolerance and plays an essential role in the formation of the immune suppression TME (Batlle and Massagué, 2019). In melanoma, the TGFβ pathway was reported to play an important role in the formation of ICI resistance (Hugo et al., 2016). In metastatic urothelial cancer, TGFβ signaling was associated with lower response rates to anti-PD-L1 (atezolizumab) therapy (Mariathasan et al., 2018). In GC, TGFβ was shown to increase the migration, adhesion and invasion of some GC cell lines but not all (Veen et al., 2021). Therefore, it is necessary to distinguish which patients could respond to therapy. There is no study correlating the TGFβ-associated signature with the TME in GC. In this study, we developed a TGFβ regulation patterns and risk scores that could predict individual patient survival outcomes and TME characteristics.

## Materials and Methods

### Data Retrieval and Preprocessing

We downloaded the RNA sequencing (RNA-seq) and survival data of TCGA-STAD (stomach adenocarcinoma) from the data portal of UCSC Xena (https://xenabrowser.net/) (Goldman et al., 2020). Then, we transformed the fragments per kilobase per million mapped fragments (FPKM) value into transcripts per kilobase million (TPM) value. We included 350 GC patients for further analysis after moving duplicates and patients without clinical information. For Gene Expression Omnibus (GEO) databases, we downloaded the expression matrices and platforms of the GSE15459 (GPL570) and GSE84437 (GPL6947) using the “GEOquery” R package. The clinical information originating from the databases that we included is summarized in Supplementary Table S1.

### Consensus Clustering

We systematically identified 54 TGFβ-pathway-related genes from the Molecular Signatures Database (MSigDB) (Supplementary Table S2). We then performed unsupervised clustering analysis to comprehensively identify two distinct TGFβ related patterns using the k-means method implemented in the “ConsensuClusterPlus” R package. We conducted 1,000 repetitions to guarantee the stability of our classification (Wilkerson and Hayes, 2010).

### Gene Set Variation Analysis

“Limma” R package was used to filtered out the differentially expressed genes (DEGs) between different TGFβ regulation patterns and then the genes were ordered by fold change (FC). The gene sets of “c5.all.v7.1.symbolsGO” and “c2.cp.kegg.v7.1.symbolsKEGG” were downloaded from MSigDB database for running GSVA analysis. Adjusted *p* with value less than 0.05 was considered as statistically significance.

### Tumor Microenvironment Cell Infiltration

The activated levels of the anticancer immune response were all downloaded from http://biocc.hrbmu.edu.cn/TIP/using the TPM value of TCGA-STAD (Chen and Mellman, 2013; Xu et al., 2018). Then, we quantified the levels of tumor-infiltrating immune cells (TIICs) using the single-sample gene-set enrichment analysis (ssGSEA) algorithm. The gene sets for calculating specific immune cells were collected from the study reported by Charoentong et al. (2017) (Supplementary Table S3) (Charoentong et al., 2017).

### Coexpression Module Networks

We conducted a network of gene coexpression to identify the TGFβ cluster-related module using the “WGCNA” R package (Langfelder and Horvath, 2008). First, we filtered out bad genes and samples using TPM data from TCGA-STAD. Then, we calculated the connection strength and developed a scale-free network using the filtered data. We examined the scale independence and modules’ average connectivity degree using the gradient method and chose the most suitable power value when setting the degree of independence as 0.85 (Chen et al., 2017). Next, using the selected power value, we generated scale-free gene coexpression networks. We regarded TGFβ clusters as a clinical factor and showed the relationship between different modules and clinical factors using heatmap. Finally, we selected the module with the closest relationship with TGFβ clusters for further analysis (Li et al., 2021).

### Creation and Validation of the Risk Score

First, we performed univariate Cox analysis on genes of the module with the closest relationship with the TGFβ clusters to screen genes having prognostic value. Then, the least absolute shrinkage and selector operation (LASSO) algorithm was used for the screened genes. Finally, using the rfsrc function in the “randomForestSRC” R package, a TGFβ-associated risk score was constructed based on the genes filtered by the LASSO algorithm. Using the median value of the risk score, GC patients were divided into high- and low-risk score groups. We developed a Kaplan-Meier survival curve, and the prognostic significance of the risk score was compared using the log-rank test. The sensitivity and specificity of the risk score for predicting survival outcome were tested by receiver operating characteristic curve (ROC) analysis using the tROC R package. The role of predicting the survival outcome of the risk score was further validated in the GSE15459 and GSE84437 databases. Univariate Cox analysis was applied to filter prognostic factors including age, sex, tumor grade, tumor stage, and risk score, and multivariate Cox analysis was further applied to filter independent prognostic factors. Moreover, a systematic nomogram was constructed based on those factors with independent prognostic values in multivariate Cox analysis.

### Statistical Analysis

Correlation coefficients between variables were computed using Pearson or Spearman correlation analyses. A t test or Mann-Whitney U test was applied to calculate the differences in continuous variables. We applied the LASSO algorithm to filter the optimal TGFβ associated genes with the best discriminative capability. Then a Kaplan-Meier survival curve was constructed and the prognostic significance of the risk score was compared using the log-rank test. The sensitivity and specificity of the risk score for predicting survival outcome were tested by ROC-curve analysis using the tROC R package. All statistical analyses were conducted with R software (version 4.0.3), and two-sided *p* < 0.05 was set as the significance criterion.

## Results

### Depicting Transforming Growth Factor *β* Clusters and Correlating Them With Tumor Microenvironment Infiltration

**FIGURE 1:**
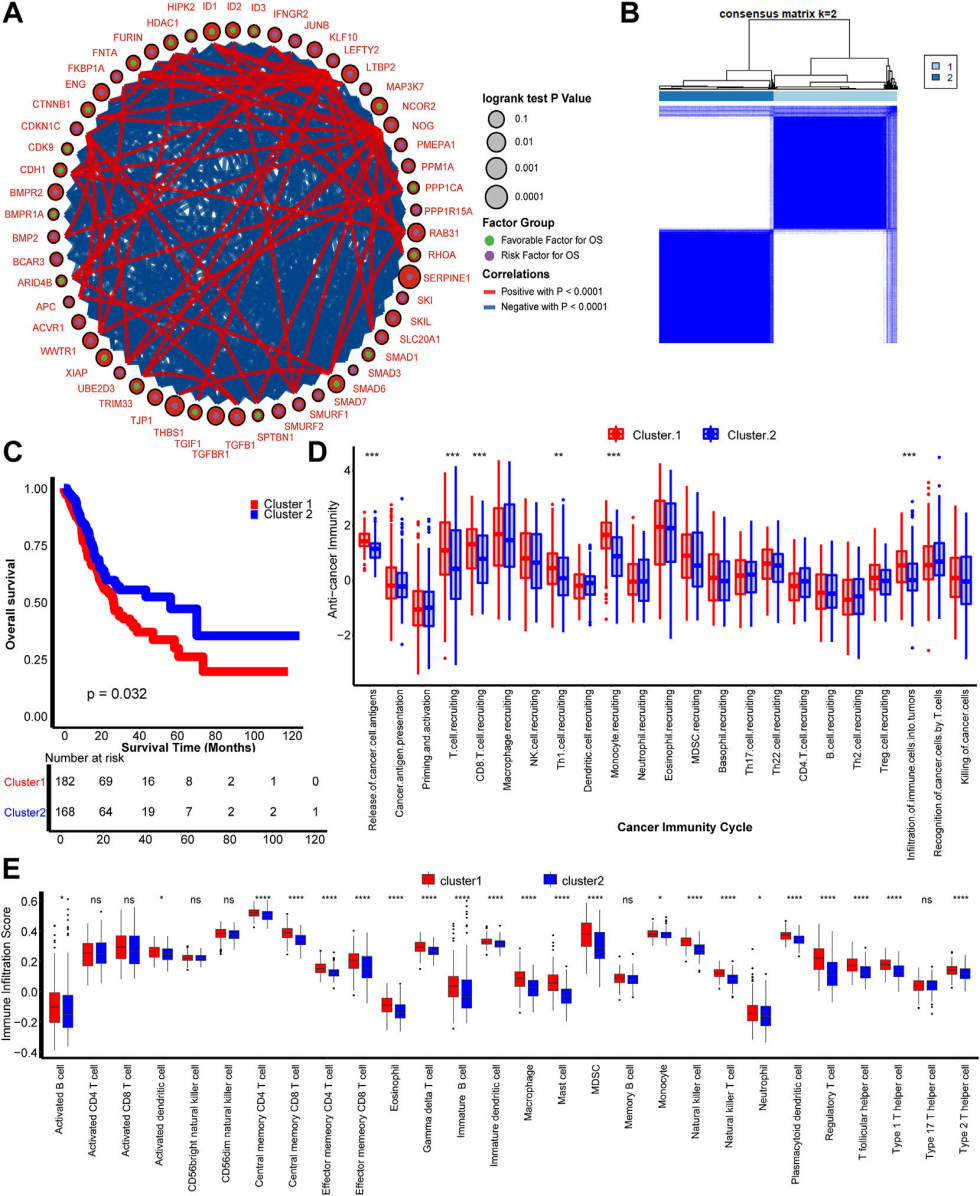
TGFβ patterns and corresponding tumor microenvironment infiltration characterization. **(A)** Fifty-four TGFβ pathway-related genes correlated with GC. Green and purple dots represent favorable and risk factors for overall survival respectively. The prognosis of each gene is expressed by the size of the circle. Curves between linked dots show the negative (blue) and positive (red) correlation between TGF pathway-related genes. **(B)** The consensus score matrix shows that all samples were grouped into clusters by the value of consensus score groups in different iterations (k = 2 in TCGA-STAD). **(C)** Survival analysis based on the TCGA-STAD dataset of TGFβ Cluster1 (red) and TGFβ Cluster 2 (blue). **(D)** Different activities of cancer immunity cycles between TGFβ Cluster 1 (red) and TGFβ Cluster 2 (blue). **(E)** TME immune cell infiltration scores between TGFβ Cluster 1 (red) and TGFβ Cluster 2 (blue). Ns, not significant; *p < 0.05; **p < 0.01; ***p < 0.001; ****p < 0.0001.

As shown in Figure 1A, most of the 54 TGFβ-pathway-related genes were prognostic factors and correlated closely with each other. We demonstrate the comprehensive landscape of gene interactions, connections, and prognostic values. Inspired by these results, we performed a comprehensive unsupervised clustering analysis based on these 54 TGFβ-pathway-related genes and two different TGFβ regulation patterns named TGFβ Cluster 1 and Cluster 2 were identified (Figure 1B, Supplementary Figures S1A–E). A total of 182 patients divided into TGFβ Cluster 1 exhibited significantly poorer survival outcomes than the other 168 patients divided into Cluster 2 (*p* = 0.032, Figure 1C).

Considering the vital role of TGFβ in shaping the immune suppression TME, we comprehensively correlated the clusters with immune phenotypes (Batlle and Massagué, 2019). The anticancer immune cycles include the following seven steps: tumor antigen release and presentation (steps 1 and 2), immune system priming and activation (step 3), transferring and invasion of immune cells to tumors (steps 4 and 5), and effector immune cells recognizing and killing cancer cells (steps 6 and 7) (Chen and Mellman, 2013). First, the relationship between TGFβ clusters and activities of anticancer immunity cycles enters our field of vision. The activities of cancer cell antigen release, T cell, CD8 T cell, T helper 1 (Th1) cell and monocyte recruitment, and infiltration of immune cells into tumors were significantly higher in TGFβ Cluster 1 than Cluster 2. These results indicate that TGFβ Cluster 1 might represent an inflamed TME subtype of GC, while TGFβ Cluster 2 represents a non-inflamed subtype (Figure 1D). Furthermore, we directly calculated the infiltration of immune cells into the TME by the ssGSEA algorithm. Consistent with the anticancer immune response, the majority of immune cells, including activated dendritic cells (DCs), effector memory CD4 T cells, effector memory CD8 T cells, macrophages, natural killer (NK) cells, had significantly higher numbers in TGFβ Cluster 1 than in Cluster 2 (Figure 1E). In addition, we found that all angiogenesis related pathways were significantly enriched in TGFβ Cluster 1, indicating a more active tumor angiogenesis in Cluster 1 (Supplementary Figure S1F, Supplementary Table S4).

### Key Modules and Hub Genes Related to Transforming Growth Factor *β* Clusters Identified by WGCNA

**FIGURE 2:**
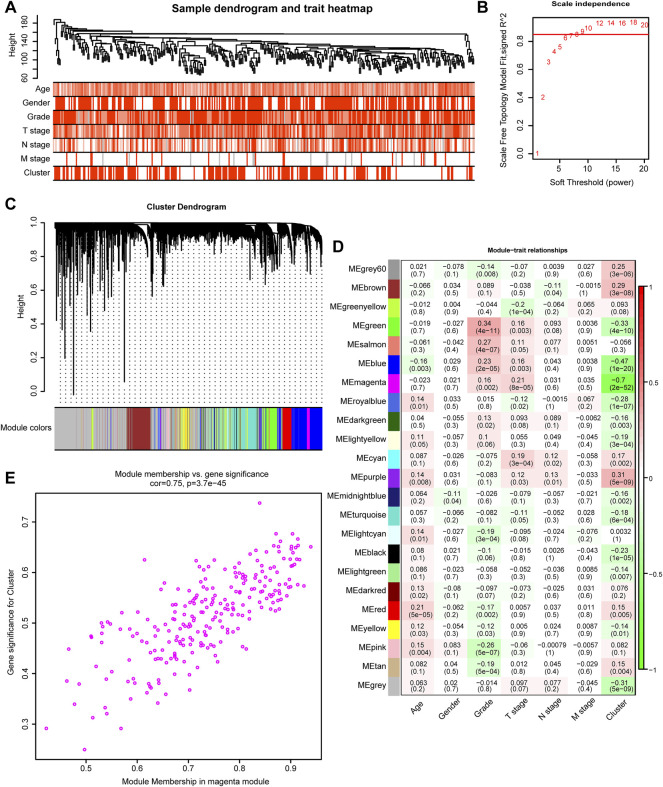
TGFβ-related modules detected by WGCNA. **(A)** Clustering dendrogram and heatmaps of clinical characteristics (age, sex, tumor stage, tumor grade, and cluster) based on 350 samples in the TCGA-STAD dataset. **(B)** Scale independence analysis by the scale-free topology model of different soft-threshold powers. **(C)** Cluster dendrogram of differentially expressed genes based on the topological overlap. **(D)** Heatmap of the correlation between various gene modules and clinical characteristics (age, sex, tumor stage, tumor grade, and cluster). Red and green represent a positive and negative correlations, respectively. **(E)** The Scatter plot shows the correlation between gene memberships in the magenta module.

We developed TGFβ based patterns that could predict survival outcomes and TME infiltration in the previous step. To predict the survival outcomes and TME infiltration of individual patients, we aimed to develop a TGFβ-based risk score. WGCNA is a method that can generate modules by similar gene expression patterns and correlate these modules with specific features, generally, clinical information (Langfelder and Horvath, 2008). In this study, we treated TGFβ clusters as clinical information and performed WGCNA combined with other clinical information including, age, sex, grade, T stage, N stage, and M stage to find the module with the closest relationship with TGFβ clusters (Figure 2A). Then, we selected 8 as the soft threshold to perform further analysis by setting the scale-free *R* ^2^ = 0.85 (Figure 2B). Twenty-three modules were filtered according to average hierarchical clustering and dynamic tree clipping (Figure 2C). The modules with the closest association with clinical information possessed the greatest biological meanings. The magenta module had the closest association with the TGFβ clusters (r = -0.70, *p* < 0.0001) (Figure 2D). In addition, the magenta module also had a close relationship with tumor grade (r = 0.16, *p* < 0.002) and T stage (r = 0.21, *p* < 0.0001) (Figure 2D). Further analysis showed that the genes in the magenta module were significantly coexpressed (cor = 0.75, *p* < 0.0001, Figure 2E, Supplementary Table S5). KEGG analysis revealed that genes could be enriched in the TGFβ pathway in the magenta module, which confirmed from another perspective that this module had a close relationship with the TGFβ pathway (Supplementary Figure S2, Supplementary Table S6). Additionally, extracellular matrix (ECM) organization, extracellular structure organization, collagen−containing extracellular matrix and ECM−receptor interaction pathways were outstanding in GO and KEGG analysis. These results indicated that the genes in the magenta module might play a critical role in the TME of GC by regulating the ECM (Supplementary Figure S2, Supplementary Table S7).

### Risk Score Development and External Validation

**FIGURE 3:**
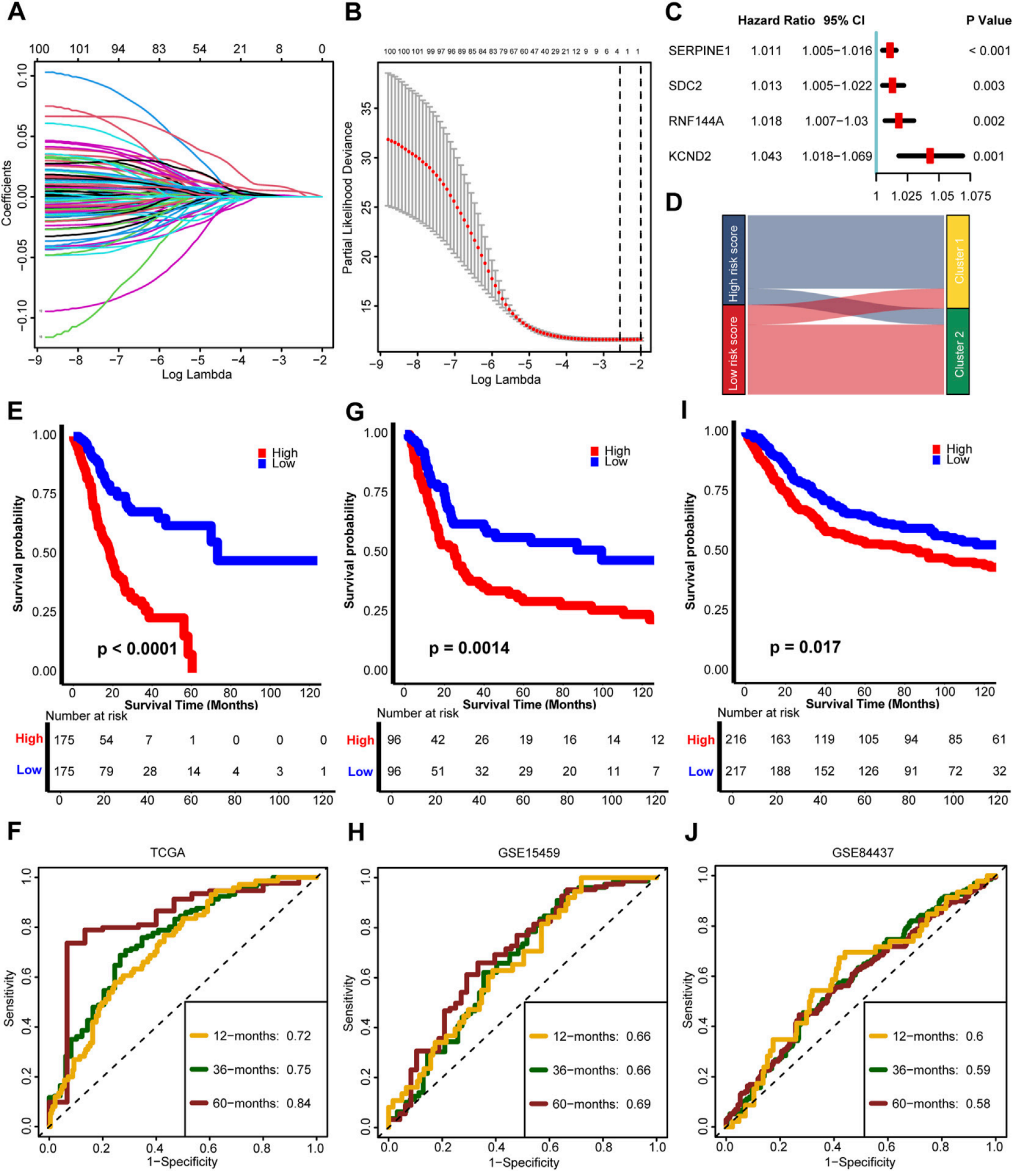
Development and external validation of a TGFβ risk score. **(A)** Least absolute shrinkage and selection operator (LASSO) coefficient curves show the log (lambda) sequence of 101 prognostic genes in the TCGA-STAD cohort. **(B)** Cross-validation for the selection of turning parameter selection by minimum criteria in the LASSO regression model. Two dotted vertical lines using the minimum criteria were plotted at the optimal values. Four genes with the best discriminative capability were selected to develop the risk score. **(C)** Univariate Cox analysis of 4 predictor genes. **(D)** The relationship between the risk score and TGFβ clusters. **(E–J)** Development and validation of the risk score in TCGA-STAD, GSE15459, and GSE84437 databases respectively and the predictive accuracy of the risk score for survival.

The 243 genes in the magenta module were selected to develop a TGFβ associated risk score. First, we filtered 101 genes with prognostic values by performing univariate Cox regression analysis (Supplementary Table S8) and identified 4 optimal candidates with minimal lambda (0.077) to generate the risk score by using random survival forest analysis (Figures 3A,B). Then we determined the prognostic values of the 4 identified genes (Figure 3C and Supplementary Table S9). By defining the median value of the risk score, we grouped the risk score into high and low risk score groups and found that high risk score group represented TGFβ Cluster1 while low risk score group represented TGFβ Cluster2 (Figure 3D). Consistent with our previous results, the higher risk score group revealed significantly poorer survival outcomes than the lower risk score group (*p* < 0.0001, Figure 3E). The predictive accuracy of the risk score for 12, 36 and 60 months was 0.72, 0.75, and 0.84 respectively (Figure 3F). Notably, we validated this risk score in external databases. Patients in the high-risk score group exhibited significantly poorer survival outcomes (*p* = 0.0014), and the predictive accuracies for 12, 36 and 60 months were 0.66, 0.66, and 0.69, respectively, in the GSE15459 cohort (Figures 3G,H). Meanwhile, in the GSE84437 cohort, a higher risk score was significantly related to poorer survival outcomes (*p* = 0.017), and the predictive accuracies for 12, 36 and 60 months were 0.60, 059, and 0.58 respectively (Figures 3I,J).

**FIGURE 4:**
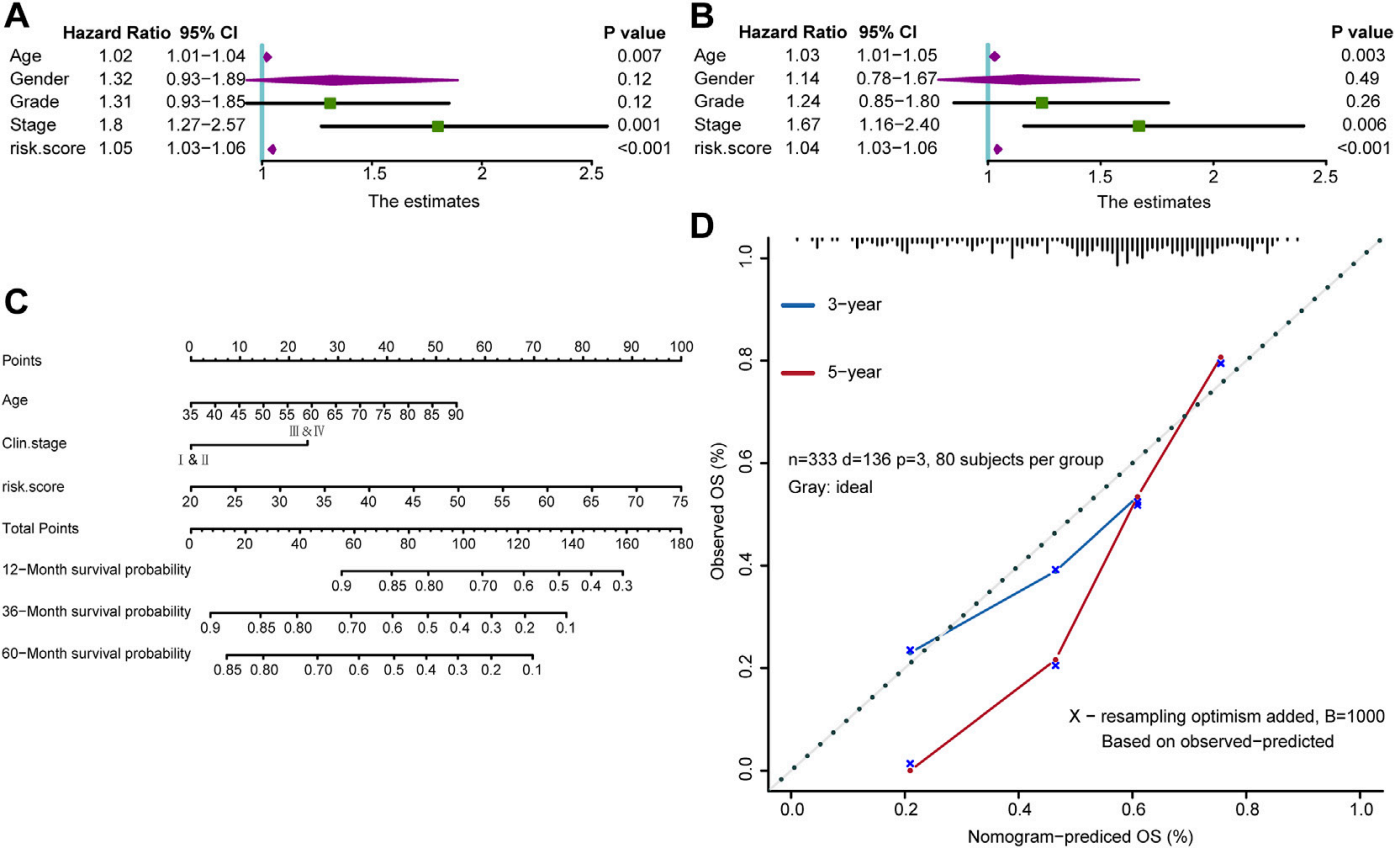
A nomogram in the TCGA-STAD cohort combined with risk score and clinicopathological characteristics. **(A,B)** Univariate and multivariate Cox analyses of the risk score and clinicopathological characteristics. **(C)** The survival outcomes at 12-month, 36-month, and 60-month were predicted using the nomogram. **(D)** Calibration curves of the nomogram were conducted using the Hosmer-Lemeshow test.

To investigate the correlation between risk score and clinical information, including age, sex, tumor grade, and TNM stage, we used univariate Cox analysis and the results showed that age, tumor stage, and risk score were all risk factors for GC (Figure 4A, Supplementary Table S10). Further multivariate Cox analysis revealed that age, tumor stage and risk score were still independent prognostic factors (Figure 4B, Supplementary Table S10). These analyses indicated that the risk score might be a potential predictive marker for the prognosis of GC. Moreover, to determine the clinical value of our findings, a nomogram was further developed by integrating the risk score, age, and tumor stage (Figure 4C). Importantly, the calibration curves showed that the predicted OS was highly consistent with the actual OS, which indicates the integrated clinical significance of this nomogram (Figure 4D).

### Associations Between Risk Score and Tumor Microenvironment Infiltration

**FIGURE 5:**
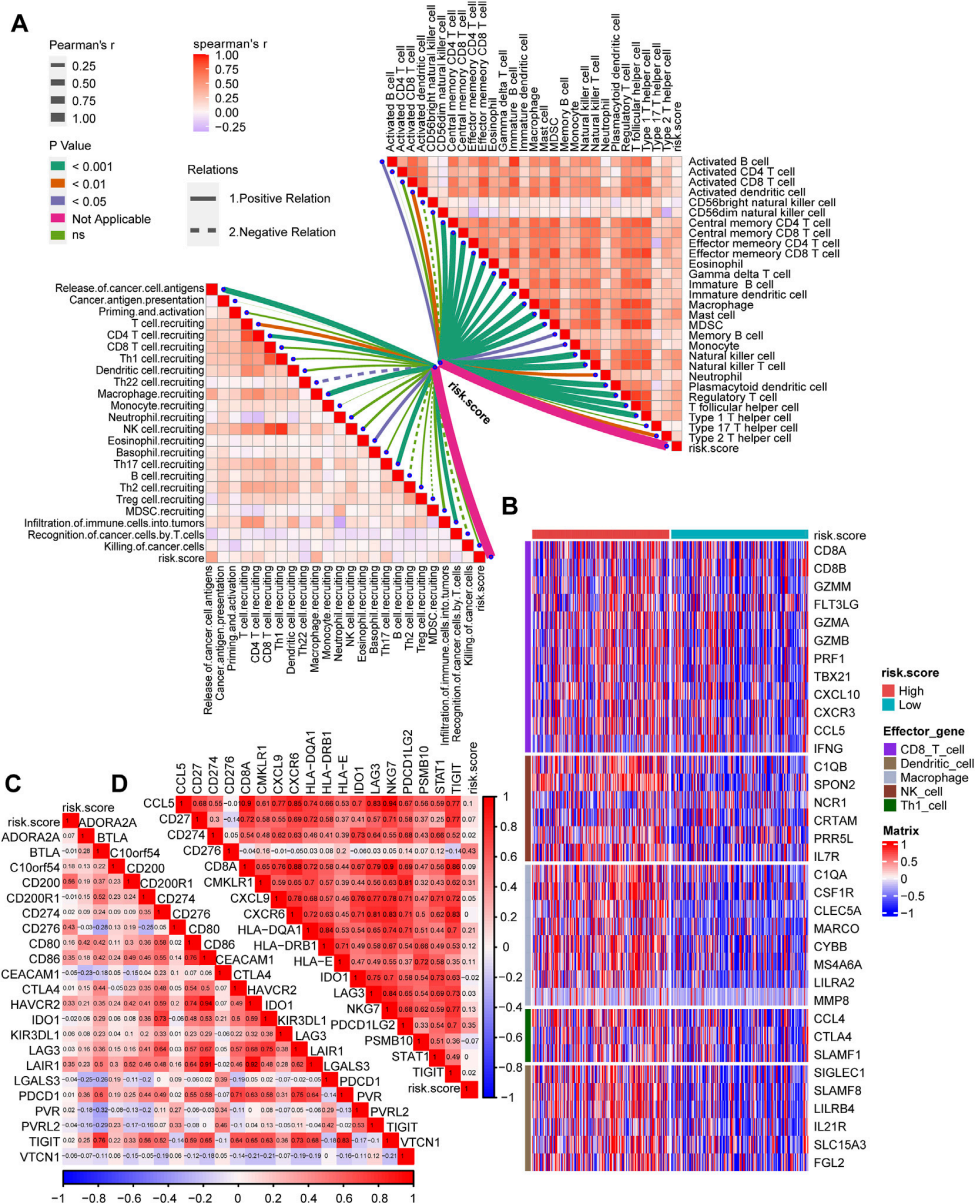
The TGFβ risk score correlated with the immune characteristics of the TME and predicted the clinical response to ICI treatment. **(A)** The correlations between the risk score and the cancer immunity cycles (left) or the infiltration of immune cells (right). **(B)** The differential expression patterns of effector genes in CD8 T cells, dendritic cells, macrophages, NK cells and Th1 cells between the high- and low-risk score groups. **(C,D)** The correlations between the risk score and immune checkpoint or the T cell inflamed gene expression.

The tumor immune microenvironment state plays a critical role in the fate of cancer cells and immunotherapy efficacy. Thus, the correlations between the risk score and the activities of cancer immunity cycles were analyzed. The release of cancer cell antigens, T cell and macrophage recruitment, infiltration of immune cells and several activities of anticancer immune responses were positively correlated with the risk score (Figure 5A, left; Supplementary Table S11). Furthermore, the infiltration of the 28 immune cells calculated using the ssGSEA algorithm, including activated DCs, central and effector memory CD4 T cells, central and effector memory CD8 T cells, NK cells and Th1 helper cells, was significantly positively correlated with the risk score (Figure 5A, right; Supplementary Table S12). Then, we divided GC patients into the high-risk score and low-risk score groups by setting the median value of the risk score as the cutoff. We determined that the higher risk score group was characterized by higher expression of CD8 T cells, DCs, macrophages, NK cells, and Th1 cells (Figure 5B). Consequently, we proposed that the inflamed phenotype in the high-risk score group may indicate a higher sensitivity to ICI treatment. Furthermore, the risk score was positively related to the expression of a majority of immune checkpoints (such as CD200, CD276, CD86, and LAIR1) (Figure 5C, Supplementary Table S13) and T cell–inflamed gene expression profiles (GEPs) (Figure 5D, Supplementary Table S14), which may indicate better ICI efficacy in GC.

## Discussion

As one of the most prominent immunosuppressive cytokines, TGFβ can regulate the generation and functions of numerous immune cells (Li and Flavell, 2008). TGFβ can activate Treg cells and attenuate T cells and DCs directly. Moreover, TGFβ can inhibit NK cells and regulate the behavior of neutrophils and macrophages. All these effects of TGFβ indicate that it plays a vital role in the formation of an immune suppression TME (Sanjabi et al., 2017). TGFβ contains 32 members of the family, which can be divided into TGFβ subfamilies and bone morphogenetic protein (BMP) subfamilies (David and Massagué, 2018). The roles of TGFβ families in gastrointestinal cancers have been widely reported. Pak et al. (2019) reported that TGFβ1 could induce the expression of VEGF-C and then promote the lymph-angiogenesis of GC (Pak et al., 2019). Li et al. (2005) reported that TGFβ1 could affect apoptosis and proliferation of GC by regulating p15 and p21 (Li et al., 2005). However, all the studies focused on only one or two members of the TGFβ families, but the comprehensive effects of TGFβ family have not been reported. In our study, we first divided GC patients into two TGFβ regulation patterns using unsupervised clustering analysis and then comprehensively analyzed the relationship between all TGFβ families. In addition, we systematically correlated these patterns with the TME of GC and developed a TGFβ based risk score to accurately predict the survival outcomes and TME characteristics in GC patients. To the best of our knowledge, our work is the first to provide TGFβ-associated prognosis and tumor infiltration characterization in gastric carcinoma.

ICI treatment has shown efficacy in numerous cancer types, including GC (Rosenberg et al., 2016; Nishino et al., 2017; Fuchs et al., 2018). However, only a minority of patients respond to the ICI treatment and the extant biomarkers are not precise enough to be used clinically (Roh et al., 2017; Fuchs et al., 2018; Panda et al., 2018). Exhaustively predictive biomarkers for ICI treatment are urgently needed to maximize the therapeutic benefit and minimize toxic side effects. An increasing number of studies support the concept that the TME plays a vital role in the ICI-based immunotherapy (Lee et al., 2014; Nishino et al., 2017). The TME comprises various cell types (endothelial cells, fibroblasts, immune cells, etc.) and extracellular components (cytokines, growth factors, hormones, extracellular matrix, etc.) that surround tumor cells and are nourished by a vascular network (Wu and Dai, 2017). Tumors characterized by immune activation and T cell infiltration are called hot tumors and have a higher response to immunotherapy (Gajewski, 2015), whereas features of T cell absence or attenuation and lower therapy-response in patients are shown in cold tumors (Liu et al., 2020a). Based on 54 TGFβ-pathway-related genes, we identified two different TGFβ regulation patterns and found that these two patterns represented inflamed and noninflamed TMEs of GC. ICI treatment might show higher response rates in the inflamed phenotype and these regulation patterns could guide precision immunotherapy treatment in GC. Moreover, we developed a TGFβ based risk score to predict an individual’s TME characteristics. It has been reported that factors involved in immune checkpoints, including PD-1, PD-L1, LAG-3, and CTLA-4, are more highly expressed in inflamed phenotypes of the TME (Gajewski et al., 2017). A majority of 28 immune checkpoint genes collected from Auslander’s study were positively correlated with the risk score (Auslander et al., 2018). Additionally, Mark et al. developed a T cell–inflamed GEP that had robust predictive value for ICI treatment (Ayers et al., 2017). In summary, we found that almost all these genes were positively correlated with the risk score based on TGFβ regulation patterns, which could be a robust predictive biomarker for the TME and might have potential value for predicting ICI treatment.

Zhang et al. (2020) assessed patterns of an RNA modification of N6-methyladenosine (m6A) and systematically correlated them with the TME infiltration characterization of GC (Zhang et al., 2020). Similar to their study and our previous study, unsupervised clustering analysis was first used to conduct a comprehensive analysis of multiple genes (Li et al., 2021). These two studies then used multiple genes to perform principal component analysis (PCA) to develop a m6A score for individual patients. In contrast, we then used the LASSO algorithm and cross validation to narrow down variables and filtered only four genes to develop a risk score in this study. Our risk score was much easier to transform to clinical application, as fewer genes needed to be detected. There are numerous gene signatures for risk stratification of GC, such as hypoxia-immune-based microenvironment gene signatures (Liu et al., 2020b), autophagy-related gene signatures (Qiu et al., 2020), metabolism-related gene signatures (Wen et al., 2020), and TP53-associated gene signatures (Nie et al., 2020). However, this is the first study developing a TGFβ-associated signature for GC. Moreover, unlike a majority of the studies reported, our study systematically correlated the risk score with TME infiltration characterization. It is worth mentioning that the area under the curve (AUC) for our risk score reached 0.84, which indicated an accurate prediction value of our risk score.

In terms of limitations, we only validated the results in several public cohorts and the relevant mechanisms of TGFβ need to be further explored *in vivo* and *in vitro*. In addition, we defined the median of the risk score as the cutoff value in all the validation cohorts, and a better cutoff value needs to be further explored for our risk score. Finally, we did not use prospective clinical trials to validate the clinical value of our risk score.

## Conclusion

We developed and validated a TGFβ-associated signature that could predict the survival outcome and TME immune characteristics of GC. Generally, this signature may aid in precision medicine for GC.

## Data Availability

The original contributions presented in the study are included in the article/Supplementary Material, further inquiries can be directed to the corresponding author.

## Supplementary Material

Supplementary Figure S1
**(A–E)** The consensus score matrix when k = 3, 4 and 5 for all samples in TCGA-STAD. **(F)** GSVA analysis shows the enrichment of angiogenesis related pathways between TGFβ clusters.

Supplementary Figure S2 GO and KEGG analysis of the TGFβ-pathway-related genes in the magenta module.

## Abbreviations

TGF, transforming growth factor; GC, gastric carcinoma; STAD, stomach adenocarcinoma; ICI, immune checkpoint inhibitor; PD-1 and PD-L1, programmed cell death-1 and its ligand; MSI, microsatellite instability; TME, tumor microenvironment; RNA-seq, RNA sequencing; FPKM, fragments per kilobase per million mapped fragments; TPM, transcripts per kilobase million; GEO, Gene Expression Omnibus; ssGSEA, single-sample gene-set enrichment analysis; LASSO, least absolute shrinkage and selector operation; ROC, receiver-operating characteristic; Th1, T helper 1; NK, natural killer; DC, dendritic cell; ECM, extracellular matrix; GEP, gene expression profiles; BMP, bone morphogenetic protein; m6A, RNA modification of N6-methyladenosine; PCA, principal component analysis; AUC, area under the curve.
